# Commentary: The thoracic nutcracker syndrome

**DOI:** 10.1016/j.xjtc.2021.05.004

**Published:** 2021-05-13

**Authors:** Sandeep Sainathan

**Affiliations:** Section of Pediatric Cardiothoracic Surgery, Department of Surgery, University of North Carolina at Chapel Hill, Chapel Hill, NC

**Central Message**

Symptomatic left bronchial compression after congenital aortic arch reconstruction surgery may need arch remodeling surgery if it cannot be adequately treated by simpler measures such as an aortopexy.

**Figure:**
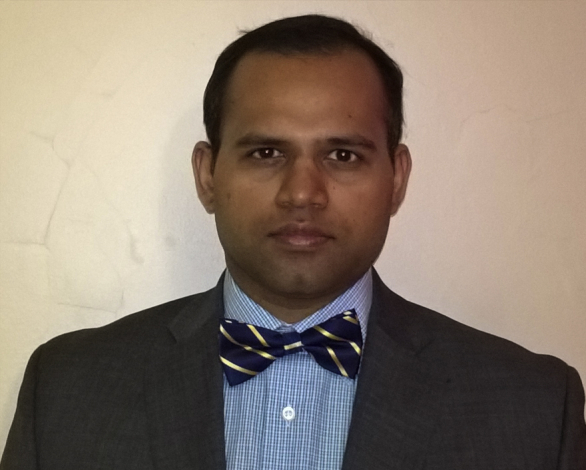
Sandeep Sainathan, MD

See Article page 126.

**Figure 1:**
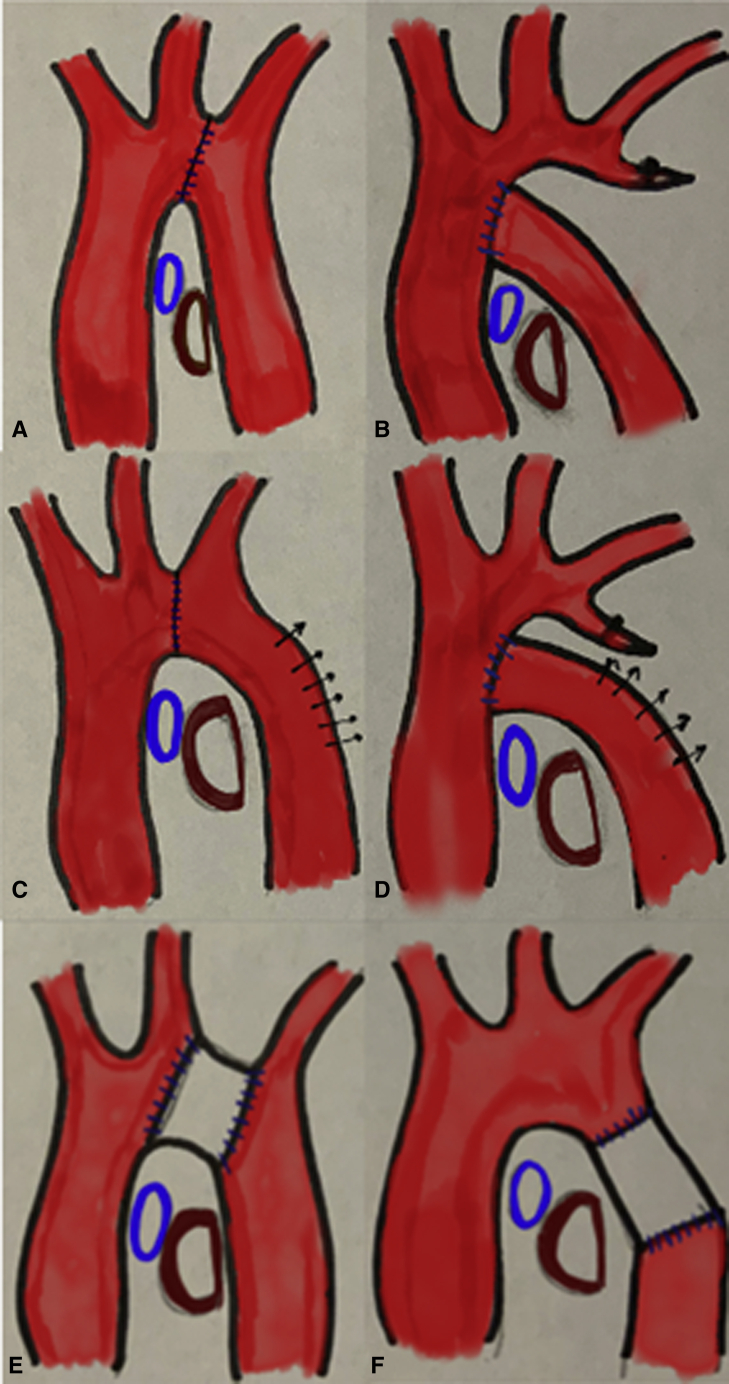
A and B, Gothic aortic arch with LMB compression after congenital aortic arch surgery. C and D, Romanesque aortic arch with relief of LMB compression after posterior aortopexy (arch remodeling). E, Crenel aortic arch after arch interposition graft relieving LMB compression. F, Romanesque aortic arch after descending aortic interposition graft relieving LMB compression. *LMB*, Left main bronchus.

Symptomatic left bronchial (LMB) compression after congenital aortic arch surgery can range from chronic cough to more challenging problems, such as recurrent pneumonia and failure to liberate from mechanical ventilation.^1^ The mechanism of compression could be due to the arch geometry. The normal aortic arch has a Romanesque shape; however, arch reconstruction surgery can induce a Gothic configuration.^2^ This leads to a nutcracker type of relation of the ascending and descending aorta with the pivot point being at the aortic arch and thus compressing the LMB (Figure 1, *A* and *B*). This can be further exacerbated if the ascending aorta is posteriorly positioned such as after a LeCompte maneuver, the descending aorta has a midline position, or the pulmonary artery is enlarged. Neonates are the most affected. In a mild case, there is potential for spontaneous improvement, particularly beyond 2 years of age due to maturation of the airway and somatic growth. The nutcracker is a type 2 lever, and its mechanical efficiency is inversely proportional to the distance between the compression point (LMB – left aortic arch) and the pivot point. Anterior and posterior aortopexy work by distracting the limbs of the nutcracker or by arch remodeling with the posterior aortopexy^3^ (Figure 1, *C* and *D*) and require a well-mobilizable aorta. Another option is to move the pivot point away from the compression point. This can be done by changing the Gothic arch to either a Crenel or a Romanesque type (Figure 1, *E* and *F*). In a case report,^4^ a Crenel arch was created by placing an aortic arch interposition graft. Chiu and colleagues^1^ in this paper describe an alternative technique of remodeling the arch to a Romanesque type by placing a proximal descending aortic interposition graft in a symptomatic patient several years after a Norwood operation. This is an attractive solution as compared with arch interposition graft, by avoiding a circulatory arrest and the risk associated with an approach through a reoperative field. As with any interposition graft in pediatrics, the main drawback is a lack of somatic growth. This they have addressed by using a novel polytetrafluoroethylene graft that can be dilated for such growth. The underlying bronchomalacia was supported with a novel off-the-shelf, on-table fashioned bespoke bioabsorbable splint.

However, concern exists for the change in the arch configuration with recurrent compression from loss of length of the interposition graft after dilation for somatic growth. The operation is a major undertaking, with cardiopulmonary bypass and risks such as paraplegia. Whether the bioabsorbable splint is replaced with a cicatrix, thus restricting future growth of the LMB, needs to be followed. Published data on airway splints have been in much younger patients in the acute setting with short-term follow-up and with other competing outcomes.^5^ Likely, such questions can only be answered when this technique is applied widely, particularly in patients with a steeper somatic growth trajectory as compared with this study's adult-size patient and with longer follow-up. Nevertheless, this case report provides an alternative technique for treating this onerous condition and may be considered when simpler measures such as an aortopexy is unsuccessful.
